# Vertebral bone quality score was associated with paraspinal muscles fat infiltration, but not modic classification in patients with chronic low back pain: a prospective cross-sectional study

**DOI:** 10.1186/s12891-024-07626-4

**Published:** 2024-07-03

**Authors:** Jiaxin Chen, Yilong Huang, Yingjuan Yang, Zhongwei Wang, Derong Zhao, Mingbin Luo, Fushun Pu, Juntao Yang, Zhenguang Zhang, Bo He

**Affiliations:** 1 Department of Medical Imaging, the First Affiliated Hospital of Kunming Medical University, Kunming, 50032 China; 2 Department of Radiology, Baoshan People’s Hospital, Baoshan, 678099 China; 3 Department of Radiology, Dali Bai Autonomous Prefecture People’s Hospital, Dali, 671099 China; 4 Department of Radiology, The First People’s Hospital of Honghe State, Mengzi, 661199 China

**Keywords:** Chronic low back pain, Fat infiltration, Vertebral bone quality score, Paraspinal muscles, Modic changes, Magnetic resonance imaging

## Abstract

**Background:**

The lumbar vertebra and paraspinal muscles play an important role in maintaining the stability of the lumbar spine. Therefore, the aim of this study was to investigate the relationship between paraspinal muscles fat infiltration and vertebral body related changes [vertebral bone quality (VBQ) score and Modic changes (MCs)] in patients with chronic low back pain (CLBP).

**Methods:**

Patients with CLBP were prospectively collected in four hospitals and all patients underwent 3.0T magnetic resonance scanning. Basic clinical information was collected, including age, sex, course of disease (COD), and body mass index (BMI). MCs were divided into 3 types based on their signal intensity on T1 and T2-weighted imaging. VBQ was obtained by midsagittal T1-weighted imaging (T1WI) and calculated using the formula: SI_L1−4_/SI_CSF_. The Proton density fat fraction (PDFF) values and cross-sectional area (CSA) of paraspinal muscles were measured on the fat fraction map from the iterative decomposition of water and fat with the echo asymmetry and least-squares estimation quantitation (IDEAL-IQ) sequences and in/out phase images at the central level of the L4/5 and L5/S1 discs.

**Results:**

This study included 476 patients with CLBP, including 189 males and 287 females. 69% had no Modic changes and 31% had Modic changes. There was no difference in CSA and PDFF for multifidus(MF) and erector spinae (ES) at both levels between Modic type I and type II, all *P* values>0.05. Spearman correlation analysis showed that VBQ was weakly negatively correlated with paraspinal muscles CSA (all r values < 0.3 and all *p* values < 0.05), moderately positive correlation with PDFF of MF at L4/5 level (r values = 0.304, *p* values<0.001) and weakly positively correlated with PDFF of other muscles (all r values<0.3 and all *p* values<0.001). Multivariate linear regression analysis showed that age (β = 0.141, *p* < 0.001), gender (β = 4.285, *p* < 0.001) and VBQ (β = 1.310, *p* = 0.001) were related to the total PDFF of muscles. For MCs, binary logistic regression showed that the odds ratio values of age, BMI and COD were 1.092, 1.082 and 1.004, respectively (all *p* values ＜  0.05).

**Conclusions:**

PDFF of paraspinal muscles was not associated with Modic classification. In addition to age and gender, PDFF of paraspinal muscles is also affected by VBQ. Age and BMI are considered risk factors for the MCs in CLBP patients.

## Introduction

Chronic low back pain(CLBP) refers to low back pain for more than 3 months [1]. It affects about 13% of adults, while also causing disability, high medical and social costs [2].

Low back pain can stem from various sources in the complex lumbar spine, including the vertebra, muscles, tendons, ligaments, fascia, facet joints, and discs [3]. Meanwhile, degenerative changes in the lumbar spine are more common in CLBP patients, such as Modic changes(MCs), cartilage endplate damage [4]. In recent years, there has been increasing attention paid to MCs as a potential source of low back pain [5]. The original classification of MCs was developed by Modic in 1988 [6]. MCs are classified into three types according to their T1-weighted and T2-weighted signal intensity(SI) on magnetic resonance imaging(MRI), Type I represents bone marrow edema and inflammation, type II represents the fatty degeneration of the bone marrow, and type III represents subchondral osteosclerosis [3, 6]. Most current studies focus on the relationship between MCs and low back pain, there are also conflicting views on the relationship between the two in different studies [7–10]. But degenerative changes in the structure of the spine are co-developed, including bone, endplate, intervertebral disc, paraspinal muscles and so on [11]. Studies have shown an association between changes in paraspinal muscles mass and CLBP-related spinal pathologies [12, 13]. However, current studies have rarely explored MCs relationship with the paraspinal muscles in CLBP patients.

In addition to the MCs of vertebral end-plate, the evaluation of the vertebral body is usually reflected by the bone mineral density(BMD) obtained by dual-energy X-ray absorptiometry (DEXA) scan or computed tomography (CT) scan [14]. A scoring system for vertebral bone quality (VBQ) based on MRI has been developed in the past few years [15] and it can protect patients from the effects of radiation [16], which has shown a correlate with bone mineral density (BMD) by using quantitative computed tomography (QCT) and has certain diagnostic efficiency [17]. Moreover, BMD has been found to be associated with paraspinal muscles fat infiltration [18].

In addition to the changes in the structure of the lumbar vertebral body, the patients with CLBP also have more obvious fat infiltration of the paraspinal muscles [12, 19]. The paraspinal muscles and the vertebral body play an important role in the stability of the lumbar spine. However, to the best of our knowledge, few studies have discussed the relationship between these two structural changes in patients with CLBP by MRI. Therefore, the purpose of this study was to investigate the relationship between paraspinal muscles fat infiltration and vertebral body related changes (VBQ score and MCs) in CLBP patients.

## Materials and methods

### Study participants

The study was approved by the institutional review board. All procedures were in accordance with the ethical standards of the 1964 Declaration of Helsinki. Verbal informed consent was obtained from all participants of the study. Participants in the study were enrolled at four multi-center healthcare facilities between July 2021 and December 2022. The patient’s gender, age, height, weight, and course of disease(COD) were collected. Body mass index(BMI) was calculated and reported in kilograms per meter square. Inclusion criteria were as follows: (1) Patients: people with CLBP (COD ≥ 3 months); (2) The age range: Non-child patients(≥ 14 years). Exclusion criteria are as follows: (1) Patients with contraindications to MR Examination and unable to cooperate with scanning; (2) Internal source of low back pain (such as urinary calculi); (3) Lumbar trauma, tumor, infection, surgery, etc.;(4) Genetic musculoskeletal diseases and neuromuscular diseases.(5) Pregnancy; (6) Athletes and regular bodybuilders who have professional muscle training; (7) Treatment(such as medications, physiotherapy) within 3 days before scan; (8) Diabetes and other chronic diseases.

### Magnetic resonance scanning

The same 3.0T MRI scanners(MR750w, GE Healthcare, Waukesha, USA) were used at all four multi-center healthcare facilities involved in the study. MRI scan sequences included routine lumbar spine sequences (Sagittal-T1WI, Sagittal-T2WI, Transverse-T2WI and Sagittal- FS T2WI), quantitative sequence(Iterative decomposition of water and fat with the echo asymmetry and least-squares estimation quantitation, IDEAL-IQ) and IDEAL sequence. The protocol was standardized across the institutions. Specific scanning parameters are listed in Table 1. An abdominal pressure band with appropriate pressure was applied to the participant’s abdomen during the scan to reduce the effect of respiratory artifacts on image quality.

**Table 1:** Magnetic resonance scanning parameter

Sequences	TE (ms)	TR (ms)	Thickness (mm)	Gaps (mm)	FOV (cm^2)^	Echo chain	Matrix	Bandwidth (KHZ)	Slices	NEX
T1WI (Sagittal)	7.3	378	4.0	1.0	32 × 32	3	320 × 224	41.67	15	3
T2WI (Sagittal)	110	2820	4.0	1.0	32 × 32	19	320 × 224	41.67	15	2
T2WI (Transverse)	110	2633	3.0	0.5	22 × 22	18	288 × 224	50	15	4
T2WI (Sagittal- FS)	90	2500	4.0	1.0	32 × 32	11	288 × 192	41.67	13	2
IDEAL-IQ (Transverse)	2.1	13.9	4.0	0	24 × 24	3	224 × 160	83.33	24	3
IDEAL (Transverse)	92.2	2000	3.0	0	20 × 20	10	288 × 192	83.3	15	6

FOV = field of view; NEX = number of excitation; T1WI = T1-weighted imaging; T2WI = T2-weighted imaging; TE = echo time; TR = repetition time; FS = fat suppression; IDEAL-IQ = iterative decomposition of water and fat with the echo asymmetry and least-squares estimation quantitation

### Image evaluation

MCs: According to the classification criteria [20], MCs are divided into 3 types, type I: hypointense on T1WI and hyperintense on T2WI, type II: hyperintense on T1WI and isointense or mildly hyperintense on T2WI, type III: hypointense on both T1WI and T2WI. The evaluation segments included L4-S1 levels.

**Fig. 1:**
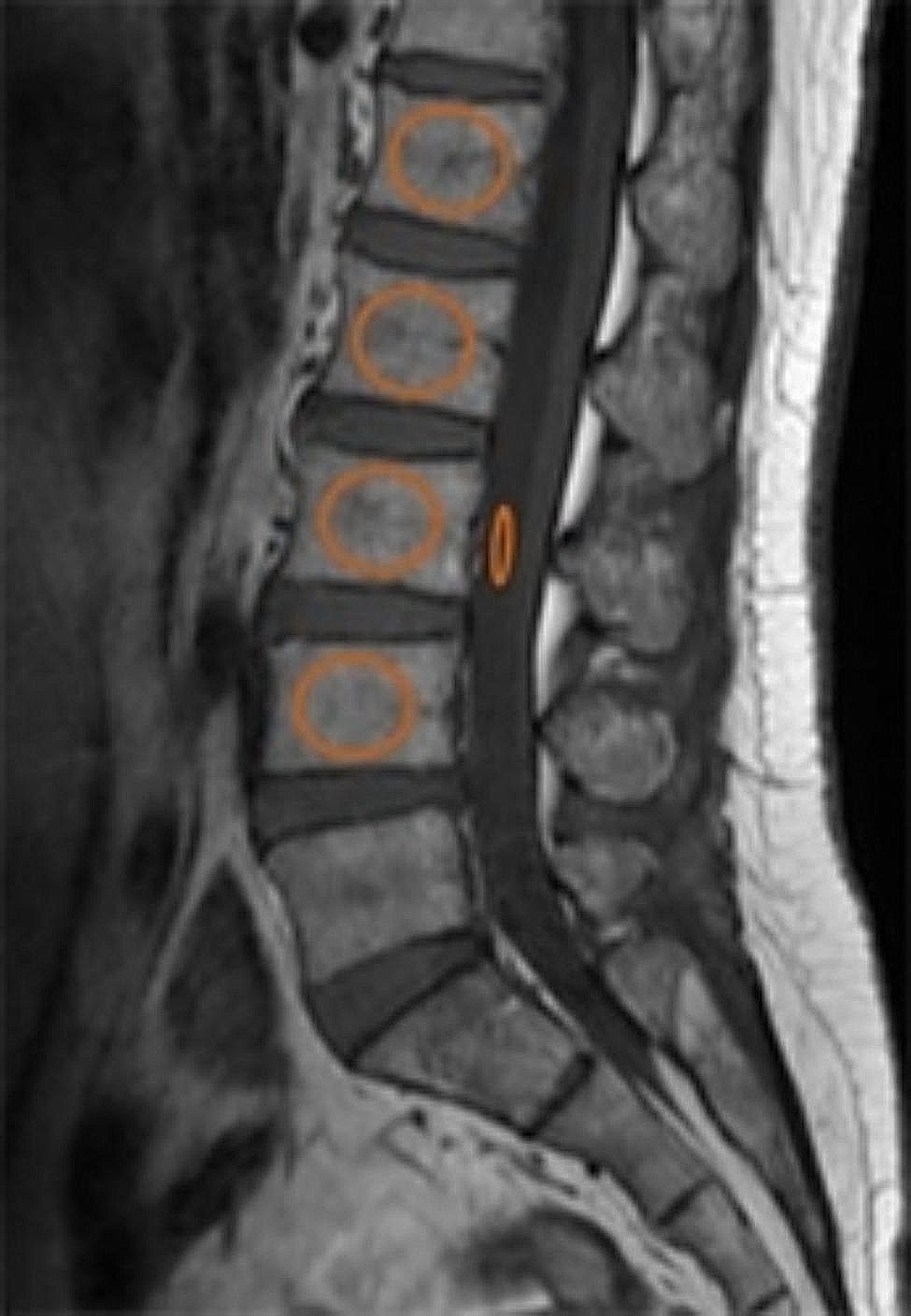
Image of a VBQ measurement. The quasi-circular ROI represents the L1-4 vertebral body, and the elliptical ROI represents the L3 level of cerebrospinal fluid; ROI = region of interest, VBQ = vertebral bone quality

Vertebral bone quality (VBQ) score: According to previous research method [21], The region of interest (ROI) was placed in the L1-4 vertebra and in the L3 level cerebrospinal fluid (CSF) in the median sagittal position(Fig. 1), and the signal intensity (SI) in each ROI was recorded. VBQ was calculated using the formula: SI_L1−4_/SI_CSF_.

**Fig. 2:**
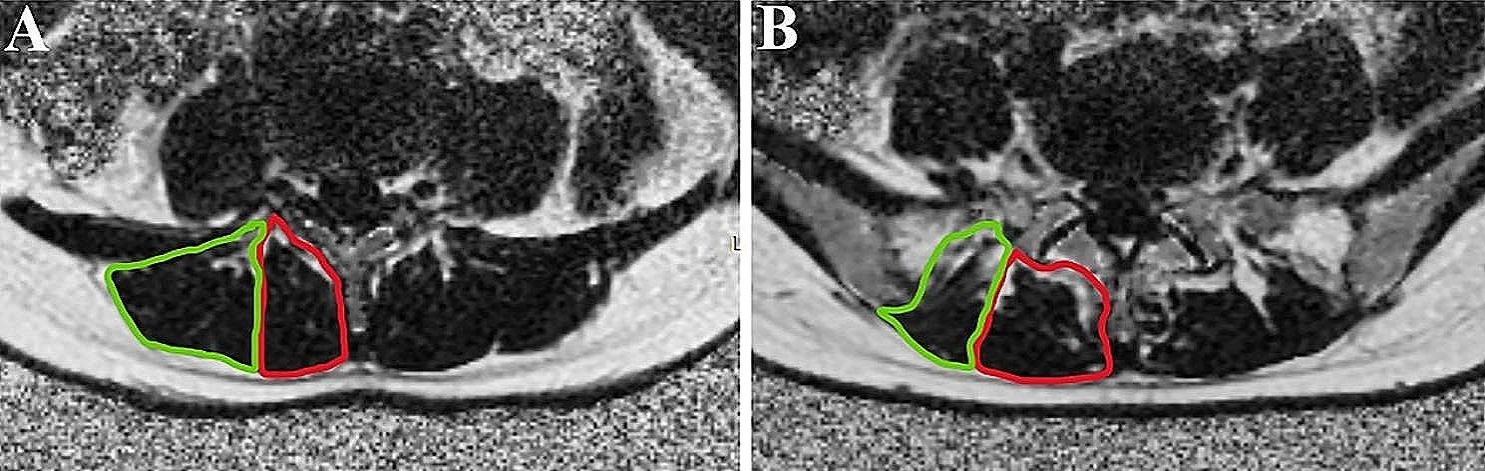
Image of a PDFF measurement. **A, B** represent the L4/5 and L5/S1 levels, respectively. Green indicates ES and red indicates MF. ES = erector spinae; MF = multifidus; PDFF = proton density fat fraction

**Fig. 3:**
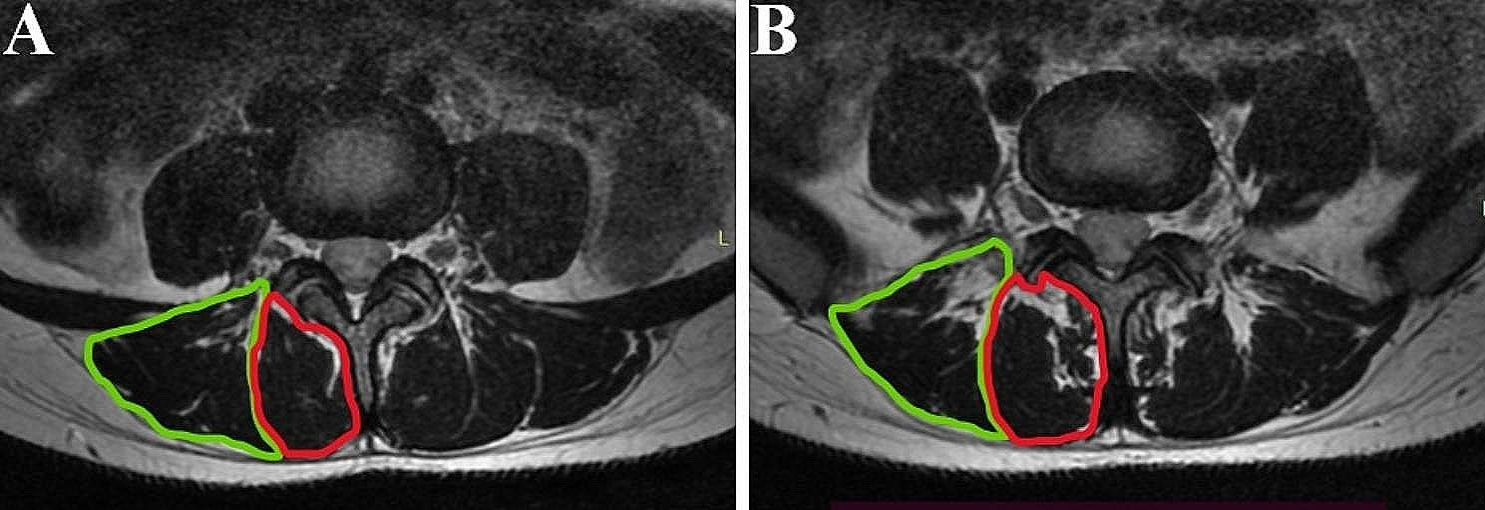
Image of a CSA measurement. **A, B** represent the L4/5 and L5/S1 levels, respectively. Green indicates ES and red indicates MF. ES = erector spinae; MF = multifidus; CSA = cross-sectional area

Paraspinal muscles proton density fat fraction (PDFF) and cross-sectional area (CSA) measurement:

The central level of the L4-S1 discs were the level of interest and the PDFF values were measured on the fat fraction map from the IDEAL-IQ sequence. Outline along the boundary of the left and right multifidus (MF) and erector spinae (ES) respectively, and finally calculate the average value of the left and right muscles(Fig. 2). We also calculated the average PDFF of a total of 8 muscles in two levels. In addition, We mapped the CSA of MF and ES in the in/out phase images from the IDEAL sequence by drawing the same way as before (Fig. 3).

Modic changes were evaluated by two experienced radiologists who were blinded to the patients’ medical records. When they do not agree, the specific Modic classification is evaluated by the superior physician(chief physician). Images of 45 patients were randomly selected for consistency test by the same personnel, who knew nothing about the previous measurement results.

### Statistical analysis

SPSS 26.0 software (IBM Corp., Armonk, NY, USA) was used for statistical analysis. Kolmogorov-Smirnov test was used to verify whether the data fit the normal distribution. The data were expressed as mean ± standard deviation or median, and the intra-class consistency test was used for data reproducibility. The t-test of two independent samples was used for the data of normal distribution, and the Mann-Whitney U test was used for the data of non-normal distribution. Spearman correlation analysis was used for correlation analysis, the r values less than 0.3 is weak, 0.3–0.6 is moderate, and greater than 0.6 is strong [22]. Multifactor linear regression analysis was used to analyze the relationship between age, gender, BMI, VBQ, COD, MCs and the total paraspinal muscles PDFF. Binary logistic regression analysis was used to explore the risk factors for MCs. As the number of Modic III types was small in this study, it was not included in the statistical analysis. *P* value < 0.05 was considered statistically significant.

## Result

### Basic information about individuals

In this study, we included a total of 476 people, including 189 males and 287 females with a median age of 48 years, a median BMI of 22.49 kg/m^2^, and a median disease course of 24 months (Table 2). Among the population in this study, 69% had no MCs and 31% had MCs, There were 67 individuals(14%) with MCs at the L4/5 level, while there were 110 individuals(23%) with MCs at the L5/S1 level (Table 2).

**Table 2:** Baseline information of cohort

Baseline characteristics	
Gender(male/ female)	189/287
Age(years)	48.00(35.00,55.00)
Hight(m)	1.62(1.58,1.68)
Wight(kg)	60.00(54.00,65.00)
BMI(kg/m^2^)	22.49(20.89,24.15)
VBQ	2.48(2.10,3.00)
COD(month)	24(12.00,60.00)
Modic changes negative(n,%)	327(69%)
Modic changes positive(n,%)	149(31%)
L4-5 positive (n,%)	67(14%)
Modic I(n,%)	40(60%)
Modic II(n,%)	27(40%)
L5-S1 positive (n,%)	110(23%)
Modic I(n,%)	52(47%)
Modic II(n,%)	58(53%)

Data are presented as median (interquartile range 25–75%) for continuous variables, counts and percentages for categorical variables. BMI = body mass index; COD = course of disease; VBQ = certebral bone quality

### Reproducibility of measurements

The intraclass coefficient of CSA was 0.911, the intraclass coefficient of PDFF was 0.847, and the intraclass coefficient of VBQ was 0.941. All *P* values were <0.05.

### Correlation analysis

Spearman correlation analysis showed that age was low negatively correlated with CSA of MF and ES at L5/S1 level(*r*=-0.099,-0.094,*p*<0.05, respectively). Age was positively correlated with PDFF(*p*<0.05) in all levels of muscles, with moderate correlation with L4/5 ES PDFF(*r* = 0.351, *p*<0.05) and weak correlation with the rest(*r*<0.3, *p*<0.05) (Table 3). There was a mild to moderate positive(*r* = 0.284–0.328,*p*<0.05) association between BMI and CSA in all levels of muscles, except for ES in L5/S1(*p*>0.05). However, for PDFF, except for a weakly positive correlation of ES at L4/5 (*r* = 0.130,*p*<0.05), the rest were not statistically significant, and there was a weakly negative correlation(*r*=-0.128, *p*<0.05) between BMI and VBQ (Table 3). The COD was negatively correlated with CSA of all levels of muscles, and positively correlated with PDFF of all levels of muscles, but there was no statistical significance(*p*>0.05), however, there was a slight positive correlation (*r* = 0.190, *p*<0.001) between the COD and VBQ (Table 3). VBQ was weakly negatively correlated with paraspinal muscles CSA(all r values<0.3 and all *p* values<0.05), moderately positive correlation with PDFF of MF at L4/5 level(r values = 0.304, *p* values<0.001) and weakly positively correlated with PDFF of other muscles(all r values<0.3 and all *p* values<0.001) (Table 3).

**Table 3:** Spearman correlation analysis

Items	L4/5MF CSA	L4/5ES CSA	L5/S1MF CSA	L5/S1ES CSA	L4/5MF PDFF	L4/5ES PDFF	L5/S1MF PDFF	L5/S1ES PDFF	Total PDFF	VBQ
Age	*r*=-0.008 *P* = 0.867	*r* = 0.030 *P* = 0.507	*r*=-0.099 *P* = 0.030	*r*=-0.094 *P* = 0.040	*r* = 0.298 *P*<0.001	*r* = 0.351 *P*<0.001	*r* = 0.221 *P*<0.001	*r* = 0.194 *P*<0.001	*r* = 0.293 *P*<0.001	*r* = 0.201 *P*<0.001
BMI	*r* = 0.300 *P*<0.001	*r* = 0.328 *P*<0.001	*r* = 0.284 *P*<0.001	*r* = 0.071 *P* = 0.121	*r* = 0.018 *P* = 0.694	*r* = 0.130 *P* = 0.005	*r* = 0.015 *P* = 0.751	*r* = 0.013 *P* = 0.785	*r* = 0.025 *P* = 0.589	*r*=-0.128 *P* = 0.005
COD	*r*=-0.005 *P* = 0.910	*r*=-0.071 *P* = 0.123	*r*=-0.013 *P* = 0.779	*r*=-0.059 *P* = 0.200	*r* = 0.061 *P* = 0.186	*r* = 0.027 *P* = 0.560	*r* = 0.047 *P* = 0.304	*r* = 0.068 *P* = 0.140	*r* = 0.064 *P* = 0.163	*r* = 0.190 *P*<0.001
VBQ	*r*=-0.091 *P* = 0.048	*r*=-0.270 *P*<0.001	*r*=-0.142 *P* = 0.002	*r*=-0.106 *P* = 0.020	*r* = 0.304 *P* = 0.186	*r* = 0.179 *P*<0.001	*r* = 0.288 *P*<0.001	*r* = 0.192 *P*<0.001	*r* = 0.270 *P*<0.001	**-** **-**

BMI = body mass index; COD = course of disease; ES = erector spinae; MF = multifidus; VBQ = vertebral bone quality; PDFF = proton density fat fraction. Bold indicates statistically significant. Bold indicates statistically significant

### Comparison of Modic type I and type II

Due to the different degrees of correlation between clinical information and lumbar spine measurement data, we first compared clinical information in order to exclude the difference between the two groups, and there was no difference in clinical information between the two groups (all *p* values>0.05). There was no difference in CSA and PDFF for ME and ES at both levels between Modic type I and type II (all *p* values>0.05) (Table 4).

**Table 4:** Comparison of muscle measurement parameters at L4/5 and L5/S1 levels Modic I versus II

Level	Type	Age (yers)	BMI (kg/m^2^)	VBQ	MF PDFF(%)	ES PDFF(%)	MF CSA (mm^2^)	ES CSA (mm^2^)
L4-5(*n* = 67)	I	55.53 ± 8.91	23.06(21.38,23.97)^a^	2.60(2.03,2.94)^a^	18.30(13.24,21.47)^a^	19.87 ± 6.91	828.51 ± 153.32	1462.81 ± 335.16
	II	56.22 ± 10.02	23.53(22.32,25.06)^a^	2.37(2.15,2.96)^a^	16.45(13.40,21.80)^a^	18.99 ± 6.97	791.99 ± 148.29	1358.07 ± 271.74
	Z/t	-0.299	-1.183	-0.026^a^	-0.051^a^	0.513	0.969	1.351
	*P*	0.766	0.237	0.980	0.959	0.610	0.336	0.181
L5/S1(*n* = 110)	I	51.79 ± 9.20	22.96(21.74,25.76)^a^	2.34(2.02,3.14)^a^	20.07(14.57,24.32)^a^	31.41 ± 9.05	990.15 ± 179.87	918.00(727.12,1195.62)^a^
	II	54.10 ± 8.90	22.59(21.35,24.29)^a^	2.47(2.21,2.89)^a^	19.70(15.85,25.56)^a^	30.65 ± 10.84	1008.99 ± 175.37	915.32(773.25,1220.37)^a^
	Z/t	-1.340	-0.838	-0.665^a^	-0.225	0.393	-0.556	-0.186^a^
	*P*	0.183	0.402	0.506	0.822	0.695	0.579	0.853

Data are presented as means (SD) and median (interquartile range 25–75%). BMI = body mass index; CSA = cross-sectional area; VBQ = vertebral bone quality; ES = erector spinae; MF = multifidus; PDFF = proton density fat fraction. ^a^ by Mann-Whitney U test

### Multifactor linear regression analysis and binary logistic regression analysis

Multivariate linear regression analysis showed that age (β = 0.141,*p*<0.001), gender (β = 4.285,*p*<0.001) and VBQ(β = 1.310,*p* = 0.001) were related to the total PDFF of muscles (Table 5). For MCs, binary logistic regression analysis showed that the odds ratio values of age, BMI and COD were 1.092, 1.082 and 1.004, respectively (all *p* values  ＜ 0.05) (Table 6).

**Table 5:** Multifactor linear regression analysis of total muscles PDFF

Variables	Total PDFF					
	β	Standered β	t	95%CI	VIF	*P*
Age	0.141	0.262	5.564	0.091–0.190	1.326	**<0.001**
Gender	4.285	0.303	7.245	3.123–5.447	1.048	**<0.001**
BMI	0.193	0.079	1.843	-0.013-0.398	1.092	0.066
COD	-0.006	0.045	-1.066	-0.016-0.005	1.080	0.287
VBQ	1.310	0.149	3.396	0.552–2.067	1.151	**0.001**
MCs	-0.553	-0.036	-0.785	-1.868-0.802	1.241	0.433

BMI = body mass index; COD = course of disease; VBQ = vertebral bone quality; VIF = variance inflation factor; MCs = modic changes; PDFF = proton density fat fraction. Bold indicates statistically significant

**Table 6:** Binary logistic regression of risk of Modic change in CLBP petients

Variables	Risk of modic change	
	OR (95% CIs)	*P*
Age	1.092(1.068–1.117)	**<0.001**
Gender	0.954(0.606–1.502)	0.839
BMI	1.082(1.002–1.170)	**0.046**
VBQ	0.747(0.555–1.007)	0.055
COD	1.004(1.000-1.008)	**0.027**

BMI = body mass index; CLBP = chronic lower back pain; COD = course of disease; VBQ = vertebral bone quality. Bold indicates statistically significant

## Discussion

In this study, we tried to explore the relationship between VBQ and MCs and paraspinal muscles in patients with CLBP. In addition, we also conducted a correlation analysis between clinical information and paraspinal muscles related parameters. We found that VBQ is associated with changes in the paraspinal muscles.

As for MCs, the current researches focused on their relationship with low back pain [7, 23]. In our study, we tried to explore the relationship between it and paraspinal muscles mass, but we found that although the PDFF of MF and ES at both levels was higher in patients with MCs type I compared with MCs type II, there was no statistical difference between them in this study. In addition, we also compared the CSA of MF and ES, and there was no statistical difference between the two types. There is currently little literature exploring the relationship between MCs and paraspinal muscles, and one study found that LBP patients with Modic type I and I/II changes had significantly higher percentage fat content compared with patients without MCs [24]. In addition, some scholars have found that at the L4/L5 and L5/S1 intervertebral disc levels, the CSA of psoas in patients with MCs is smaller than that in patients without these changes [25]. However, those studies did not explore the differences between Modic type I and II. The MCs of type I and type II are the most common patterns observed in the lumbar spine [7]. In this study, the incidence of MCs was 31%, and the amount of Modic type III was small, so it was not included in the statistical analysis. In addition, Other scholars have found that age is associated with MCs in the lower lumbar region(L4-S1) [26], and the results of this study are consistent with it, and we found that age is a risk factor(OR = 1.092). Other scholars have also pointed out that MCs are related to weight-related factors (BMI, waist circumference) [27], and this study also has similar finding. Binary logistic regression analysis showed that the OR of age, BMI and COD were all greater than 1, and the *P*-values were all less than 0.05, but the OR values were too small to have clinical value, which may be caused by numerous factors affecting MCs.

VBQ is based on MRI, which has been used to measure fatty infiltration within the vertebral body [15, 28]. In this study, it was found by multiple regression that there was a relationship between VBQ and total paraspinal muscles PDFF, and the PDFF increased by 1.31 units for every unit increase of VBQ. Through correlation analysis, we also found that VBQ was weakly negatively correlated with paraspinal muscles CSA, and was weakly positively correlated with PDFF of other muscles except for moderately positive correlation with PDFF of MF at L4/5 level. To our knowledge, there were few studies to explore the association of VBQ with paraspinal muscles fat infiltration and the specific causal relationship requires further longitudinal study. However, several studies have found that lower BMD may be associated with more severe paraspinal muscles fatty infiltration [18, 29, 30]and these studies may provide some reference value to our result. We also found a weak positive correlation between VBQ and age, which may indicate that the vertebral body has more fat with increased age. Interestingly, we observed a weak negative correlation between BMI and VBQ. Generally speaking, BMI and VBQ are both parameters that reflect fat to some extent, and there should be a positive linear relationship between the two. However, BMI reflects the general fat content and can’t accurately reflect the fat situation of the tissue [31], while VBQ mainly reflects the fat content of the vertebral body [28]. This may explain the relationship between the two in this study.

It has been found that the CSA of paraspinal muscles decreases with age [32, 33], this is similar to the results in our study that we found a very weak negative correlation between age and CSA of MF and ES at the L5/S1 level. In addition to the influence of age on paraspinal muscles CSA, the present studies also confirmed that age also affects paraspinal muscles fat infiltration [32, 34], which is similar to our results. We also found a low to moderate positive correlation between BMI and most paraspinal muscles’ CSA,

and only a weak positive correlation with PDFF of ES at the L4/5 level, a study of paraspinal and lower limb muscles also found that BMI was not associated with fat infiltration in paraspinal muscles [35], and it has been pointed out that BMI does not estimate the amount of fat in different parts of the body [31], which may be the reason why there was less correlation between BMI and paraspinal muscles PDFF in this study. We did not find a relationship between the COD and paraspinal PDFF, which may be due to the large span of the course of the disease in this study and the lack of detailed records of patients’ low back pain, whether it was persistent or intermittent. Finally, through multi-factor regression, we found that age and gender also had an impact on the PDFF of paraspinal muscles, which was similar to the results of Huang Y and Huang R et al [12, 32]. Of course, in order to exclude the influence of potential factors on paraspinal muscles’ PDFF, we also excluded patients with chronic diseases such as diabetes. Because diabetes is a chronic metabolic disease, which has been reported individuals with impaired glucose metabolism showed significantly higher PDFF of muscles compared with controls [36, 37].

There are some limitations to our study. First of all, because the amount of Modic III was very small in this study, statistical analysis was not included. Secondly, based on the mismatch of clinical information, we did not compare the relevant parameters of the population with and without MCs. Finally, in this study, we selected the L4-S1 levels as the level of interest, which could not represent the entire lumbar spine levels, but studies have shown that MCs and paraspinal muscles fat infiltration are more obvious at the lower lumbar spine level [26, 38].

## Conclusion

There is no changes in paraspinal muscles’ CSA and PDFF between Modic type I and type II. VBQ has a positive linear relationship with total paraspinal muscles’ PDFF. In addition to age and gender, total paraspinal muscles’ PDFF is also affected by VBQ. Age has a weak negative correlation with paraspinal muscles CSA, a low to moderate positive correlation with PDFF, and a low positive correlation with VBQ. Overall, BMI had a low to moderate positive correlation with paraspinal muscles CSA, but no correlation with PDFF. Age and BMI are considered risk factors for the MCs in CLBP patients.

## Data Availability

The datasets used and/or analysed during the current study are available from the corresponding author on reasonable request.

## Abbreviations

BMD: Bone mineral density
BMI: Body mass index
CT: Computed tomography
CLBP: Chronic low back pain
COD: Course of disease
CSA: Cross-sectional area
CSF: Cerebrospinal fluid
DXA: Dual-energy X-ray absorptiometry
ES: Erector spinae
MF: Multifidus
IDEAL-IQ: Iterative decomposition of water and fat with the echo asymmetry and least-squares estimation quantitation
MCs: Modic changes
MRI: Magnetic resonance imaging
PDFF: Proton density fat fraction
QCT: Quantitative computed tomography
ROI: Region of interest
SI: Signal intensity
VBQ: Vertebral bone quality
